# Glucagon-like peptide-1 prevents methylglyoxal-induced apoptosis of beta cells through improving mitochondrial function and suppressing prolonged AMPK activation

**DOI:** 10.1038/srep23403

**Published:** 2016-03-21

**Authors:** Tien-Jyun Chang, Hsing-Chi Tseng, Meng-Wei Liu, Yi-Cheng Chang, Meng-Lun Hsieh, Lee-Ming Chuang

**Affiliations:** 1 Department of Internal Medicine, National Taiwan University Hospital, Taipei 10002, Taiwan; 2 Institute of Molecular Medicine, National Taiwan University Medical College, Taipei 10002, Taiwan; 3 Graduate Institute of Medical Genomics and Proteomics, National Taiwan University Medical College, Taipei 10002, Taiwan; 4 Institute of Biomedical Science, Academia Sinica, Taipei, 11500, Taiwan

## Abstract

Accumulation of methylglyoxal (MG) contributes to glucotoxicity and mediates beta cell apoptosis. The molecular mechanism by which GLP-1 protects MG-induced beta cell apoptosis remains unclear. Metformin is a first-line drug for treating type 2 diabetes associated with AMPK activation. However, whether metformin prevents MG-induced beta cell apoptosis is controversial. Here, we explored the signaling pathway involved in the anti-apoptotic effect of GLP-1, and investigated whether metformin had an anti-apoptotic effect on beta cells. MG treatment induced apoptosis of beta cells, impaired mitochondrial function, and prolonged activation of AMP-dependent protein kinase (AMPK). The MG-induced pro-apoptotic effects were abolished by an AMPK inhibitor. Pretreatment of GLP-1 reversed MG-induced apoptosis, and mitochondrial dysfunction, and suppressed prolonged AMPK activation. Pretreatment of GLP-1 reversed AMPK activator 5-aminoimidazole-4-carboxamide riboside (AICAR)-induced apoptosis, and suppressed prolonged AMPK activation. However, metformin neither leads to beta cell apoptosis nor ameliorates MG-induced beta cell apoptosis. In parallel, GLP-1 also prevents MG-induced beta cell apoptosis through PKA and PI3K-dependent pathway. In conclusion, these data indicates GLP-1 but not metformin protects MG-induced beta cell apoptosis through improving mitochondrial function, and alleviating the prolonged AMPK activation. Whether adding GLP-1 to metformin provides better beta cell survival and delays disease progression remains to be validated.

Beta cell apoptosis is one of the crucial etiologies of diabetes^1^. Chronic hyperglycemia leads to the formation of advanced glycation end-product (AGEs) through promoting non-enzymatic glycation of endogenous proteins, lipids and nucleic acids^2^. Methylglyoxal (MG) is one kind of intracellularly formed α-ketoaldehydes, which are essential sources of intracellular AGEs. Abnormal accumulation of MG has been implicated in causing damage in various tissues and organs^3^. MG causes apoptosis in a dose-dependent manner in RINm5F cells and other rat pancreatic beta cells^4^. Chronic infusion of MG induces type 2 diabetes and MG is considered as a possible mediator of hyperglycemia-induced type 2 diabetes^5^. MG also suppresses insulin secretion and leads to apoptosis in rat pancreatic beta cells^5^.

Glucagon-like peptide 1 (GLP-1) is an incretin hormone with 30 amino-acid secreted by duodenal L-cells. GLP-1 enhances proliferation and inhibits apoptosis of pancreatic beta cells with beneficial effects on beta cell mass. The anti-apoptotic effects of GLP-1 have been found in animal models and in cultured pancreatic beta cell lines^6^,^7^. GLP-1 also counteracts the pro-apoptotic effects of streptozotocin^8^, hydrogen peroxide^6^, fatty acids, and cytokines^9^. The GLP-1 receptor agonist, exendin-4, ameliorates human islet amyloid polypeptide-induced beta cell death partially through the activation of the Akt pathway and enhanced mitochondrial biogenesis^10^. Exendin-4 also rescues the cytokine-induced reduction of electron transport chain proteins of mitochondria and leads to decrease oxidative stress and alleviate apoptosis^11^.

Sharma *et al.* recently reported the GLP-1 analogue liraglutide ameliorates MG-induced cytotoxicity and apoptosis in human neuroblastoma cell SH-SY5Y through enhanced expression of pro-survival Mcl1 signaling protein, activation of Akt, MEK1/2, and transcription factor p90RSK^12^. Kimura *et al.* also reported that the neuroprotective effects of GLP-1 on reducing MG-induced apoptosis are through transactivation of EGFR and subsequent PI3K/Akt/mTOR/GCLc/redox pathway in PC12 cells^13^. However, whether and how GLP-1 receptor agonist rescues MG-induced apoptosis of pancreatic beta cells has not been reported. In this study, we demonstrated the potential effect of the GLP-1 receptor agonist on MG-induced beta cell apoptosis and investigated the underlying molecular mechanisms.

Metformin is a first-line drug for treating type 2 diabetes^14^. It is well known that the pleiotropic actions of metformin are associated with activation of AMP-activated protein kinase (AMPK)^15^. Metformin has been reported to protect human islets against lipotoxicity^16^. On the other hand, metformin has also been reported to prevent human pancreatic islets from high glucose-induced impairment of glucose-stimulated insulin secretion (GSIS)^17^. However, the effect of metformin on MG-induced beta cell apoptosis is not clear. In this study, we investigated whether metformin had an anti-apoptotic effect on beta cells.

## Results

### GLP-1 protects beta cells from MG-induced apoptosis

**Figure 1:**
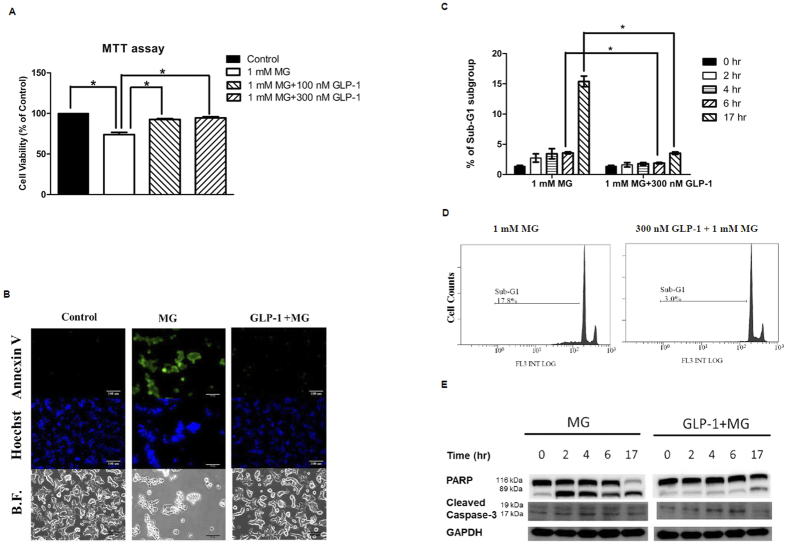
GLP-1 protects rat insulinoma cells RINm5F from MG-induced apoptosis. RINm5F Cells were treated in the presence or absence of 1 mM MG with or without GLP-1 (100 nM or 300 nM). (**A)** Cell viability was measured by MTT assay. Data are shown as relative cell viability (mean % ± S.E. bar) as compared with that in control (n = 4). *p < 0.05. (**B)** Apoptosis was demonstrated by Annexin V/ Hoechst 33342 staining after incubated with indicated treatment for 17 hr. Annexin V positive cells showed green fluorescence (upper row). Condense nuclei were shown in apoptotic cells by Hoechst 33342 staining (middle row). The pictures on bright field were shown in the lower row. (**C)** The percentage of apoptotic cells was calculated by measuring the percentage of cells in the sub-G1 population in the indicated time by using flow cytometry with propidium iodide (PI) staining (n = 3). *p < 0.05. (**D)** The cell counts and percentage of apoptotic cells in the sub-G1 population after incubation with indicated treatment for 17 hr were measured by using flow cytometry with propidium iodide (PI) staining. (**E)** Western blot of poly(ADP-ribose) polymerase (PARP) and cleaved caspase-3. The positions of the 113 kDa and 89 kDa in Western blot represent intact PARP protein and its cleavage products, respectively. The positions of the 19 kDa and 17 kDa in Western blot represent cleaved caspase-3. GAPDH was used as an internal control.

We performed 3-(4,5-dimethylthiazol-2-yl)-2,5-diphenyltetrazolium bromide (MTT) assay to analyze cell survival with 1 mM MG incubated for 17 hr. The cell survival rate decreased to about 70% that of the control. If the cells were pretreated with 100 or 300 nM GLP-1, the cell survival rate was nearly the same as that of the control (Fig. 1A). This indicated that GLP-1 can prevent MG-induced cytotoxicity in beta cells.

We next performed annexin V and Hoechst staining after incubated with indicated treatment for 17 hr to specifically determine the proportion of cell apoptosis. Most of the MG-treated RINm5F cells were stained with annexin V (green, Fig. 1B, upper middle image). Pretreatment with GLP-1 reduced annexin V staining (green, Fig. 1B, upper right image) to an extent comparable to that of the control cells (green, Fig. 1B, upper left image). Moreover, nuclear staining with Hoechst 33342 demonstrated apparent nuclear condensation of apoptotic cells induced by MG (Fig. 1B, middle image), but not in GLP-1 pretreated cells (Fig. 1B, middle right image). On a bright field, an apparently morphological change to a round shape was found in most MG-treated cells (Fig. 1B, lower middle image), while GLP-1 pretreated cells were maintained a patchy epithelial morphology (Fig. 1B, lower right image).

Annexin V/Hoechst staining could not provide a quantitative result because RINm5F cells easily stacked with each other. Therefore, we analyzed a sub-G1 cell population using flow cytometry with PI staining to calculate the percentage of apoptotic cells. After 4 h and 6 h, the percentage of sub-G1 cell population was 2 times higher in the MG-treated cells than in the GLP-1 pretreated cells (Fig. 1C). More prominently, when RINm5F cells were treated with 1 mM MG for 17 hr, the percentage of sub-G1 cell population was approximately 5 times higher than that of the GLP-1 pretreated cells (Fig. 1C,D).

In mammalian cells, the caspase-3 is a 32 kDa inactive precursor requiring cleavage at specific aspartate residues to be converted into an active protease when cells receive the signal of apoptosis^18^. One of the substrates for caspase-3 during apoptosis is the 116 kDa poly(ADP-ribose) polymerase (PARP). Apoptosis is characterized by activation of caspase-3 and subsequent cleavage of PARP into 89- and 24-kDa fragments^18^. The positions of the 116-kDa and 89-kDa in Western blot represent intact PARP protein and its cleavage product, respectively (Fig. 1E). The positions of cleavage form of caspase-3 in Western blot were 19 and 17 kDa (Fig. 1E). MG activated caspase-3 and the cleavage of PARP at 2 h and this effect persisted for 17 hr. However, pretreatment with GLP-1 down-regulated MG-induced activation of caspase-3 and the cleavage of PARP in RINm5F cells (Fig. 1E).

### GLP-1 suppressed MG-induced beta cell apoptosis through protein kinase A (PKA) and PI3K dependent pathway, respectively

**Figure 2:**
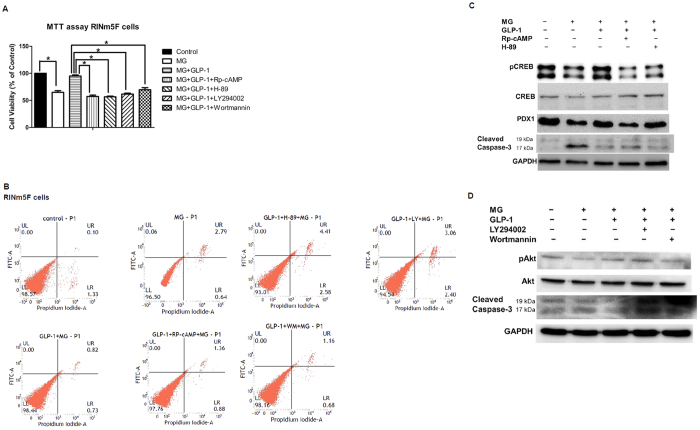
GLP-1 suppressed MG-induced beta cell apoptosis through protein kinase A (PKA) and PI3K dependent pathway, respectively. RINm5F cells were treated in the absence of MG, 1 mM MG, 1 mM MG + 300 nM GLP-1, 1 mM MG + 300 nM GLP-1 + 100 μM Rp-cAMP, 1 mM MG + 300 nM GLP-1 + 10 μM H-89, 1 mM MG + 300 nM GLP-1 + 30 μM LY294002, 1 mM MG + 300 nM GLP-1 + 50 nM wortmannin, respectively. **(A)** Cell viability was measured by MTT assay. Data are shown as relative cell viability (mean % ± S.E. bar) as compared with that in control (n = 3). *p < 0.05. **(B)** Annexin-V/PI flow cytometry. LL: left lower quadrant indicated viable cells, UL: upper left quadrant indicated early apoptotic cells, UR: upper right quadrant indicated late apoptotic cells, LR: right lower quadrant indicated necrotic cells. **(C)** Western blot of pCREB/CREB, PDX1, cleaved caspase-3, and GAPDH was used as an internal control. **(D)** Western blot of pAkt/Akt, cleaved caspase-3, and GAPDH was used as an internal control.

It has been reported that activation of GLP-1 receptor by exendin-4 inhibits hydrogen peroxide-induced apoptosis of MIN6 cells in a cAMP- and PI3K-dependent manner with upregulation of Bcl-2/Bcl-xL and downregulation of PARP^19^. GLP-1 also activates Akt/PKB and prevents apoptosis of INS-1 cells in response to glucolipotoxicity or staurosporin^20^,^21^. In this study, we found both PKA inhibitors (Rp-cAMP and H-89) and PI3K inhibitors (LY294002 and wortmannin) abrogated the anti-apoptotic effect of GLP-1 in RINm5F cells based on the results of MTT assay (Fig. 2A). Furthermore, we applied Annexin-V/PI flow cytometry to differentiate and quantitatively determine viable cells [LL quadrant: Annexin V (−), PI (−)], early apoptotic cells (UL quadrant, Annexin V (+), PI (−)], late apoptotic cells [UR quadrant, Annexin V (+), PI (+)], and necrotic cells [LR quadrant, Annexin V (−), PI (+)]. In Fig. 2B, MG induced cell apoptosis (0.06% early apoptosis, 2.79% late apoptosis), and pretreatment of GLP-1 rescued MG-induced cell apoptosis (0% early apoptosis, 0.82% late apoptosis). When PKA inhibitors were added, the anti-apoptotic effect of GLP-1 was abolished (GLP-1 + Rp-cAMP + MG: 0% early apoptosis, 1.36% late apoptosis; GLP-1 + H-89 + MG: 0% early apoptosis, 4.41% late apoptosis). When PI3 kinase inhibitors were added, the anti-apoptotic effect of GLP-1 was also abolished (GLP-1 + LY294002 + MG: 0% early apoptosis, 3.06% late apoptosis; GLP-1 + wortmannin + MG: 0% early apoptosis, 1.16% late apoptosis).

We further explored the well-established PKA and PI3K signaling pathway of GLP-1. Treatment with MG suppressed phosphorylation of CREB and PDX1 expression with activation of caspase-3, and pretreatment with GLP-1 restored the phosphorylation of CREB and PDX1 expression with inhibition of caspase-3 (Fig. 2C). When the PKA inhibitors (Rp-cAMP and H-89) were added, the phosphorylation of CREB and PDX1 expression were suppressed with partially activated caspase 3 (Fig. 2C). Treatment with MG inhibited phosphorylation of Akt with activation of caspase-3, and pretreatment with GLP-1 restored the phosphorylation of Akt with suppressed caspase-3 activation. When the PI3K inhibitors (LY2940002 and wortmannin) were added, the phosphorylation of Akt was partially inhibited with activation of caspase-3 (Fig. 2D).

### GLP-1 ameliorated MG-induced mitochondrial dysfunction, ATP depletion, and suppressed prolonged AMPK activation

**Figure 3:**
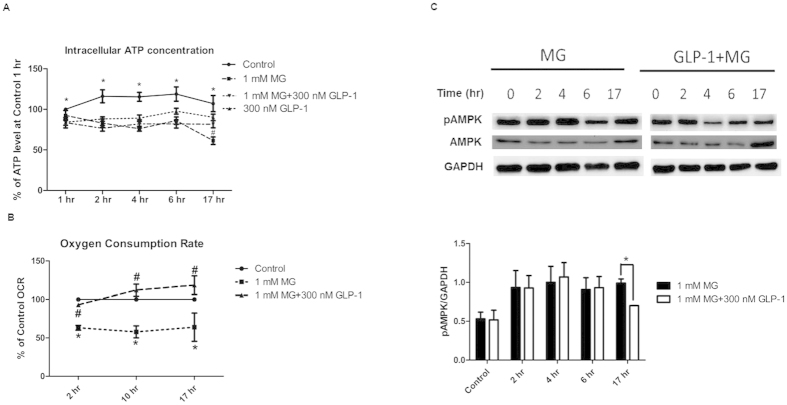
GLP-1 rescued MG-induced mitochondria dysfunction and inhibited prolonged AMPK activation in RINm5F cells. Cells were treated in the absence or presence of 1 mM MG with or without 300 nM GLP-1 for indicated time. (**A)** Relative intracellular ATP concentration (%) compared with control in indicated time (n = 6). *p < 0.05 (Control vs. 1 mM MG at 1 hr, 2 hr, 4 hr, 6 hr and 17 hr), #p < 0.05 (1 mM MG vs. 1 mM MG + 300 nM GLP-1 at 17 hr) (**B)** Relative oxygen consumption rate (%) compared with control in indicated time (n = 3). *p < 0.05 (Control vs. 1 mM MG at 2 hr, 10 hr, and 17 hr), #p < 0.05 (1 mM MG vs. 1 mM MG + 300 nM GLP-1 at 2 hr, 10 hr, and 17 hr). (**C)** Western blot of p-AMPK/AMPK. GAPDH was used as internal control. The bar graph showed the ratio of pAMPK/GAPDH in Western blot in the absence or presence of 1 mM MG with or without 300 nM GLP-1 in the indicated time (n = 3). *p < 0.05.

Steady decline in ATP production was observed in MG-treated cells (Fig. 3A). Pretreatment of GLP-1 partially rescued ATP production after 17 h treatment with MG (Fig. 3A). To monitor in real time mitochondrial respiration of RINm5F cells during different treatments, we used a Seahorse XF analyzer to measure oxygen consumption rate. A ~40% reduction in oxygen consumption rate was found in the RINm5F cells from 2 h till 17 h during MG treatment (Fig. 3B). In cells pretreated with GLP-1, the oxygen consumption rate was almost totally recovered (Fig. 3B).

Reduction of the intracellular ATP/AMP ratio has been shown to activate AMPK through phosphorylation of threonine 172 of α-subunit^22^. The role of AMPK on beta cell survival is still controversial. Therefore, we further explored the effects of MG and GLP-1 on the activity of AMPK. Consistent with the reduced ATP production observed in MG-treated cells, prolonged activation of AMPK was observed in MG-treated cells. Pretreatment with GLP-1 reduced AMPK phosphorylation in MG-treated cells, especially at 17 hr. (Fig. 3C).

### AMPK inhibitor partially prevents MG-induced cell death and apoptosis

**Figure 4:**
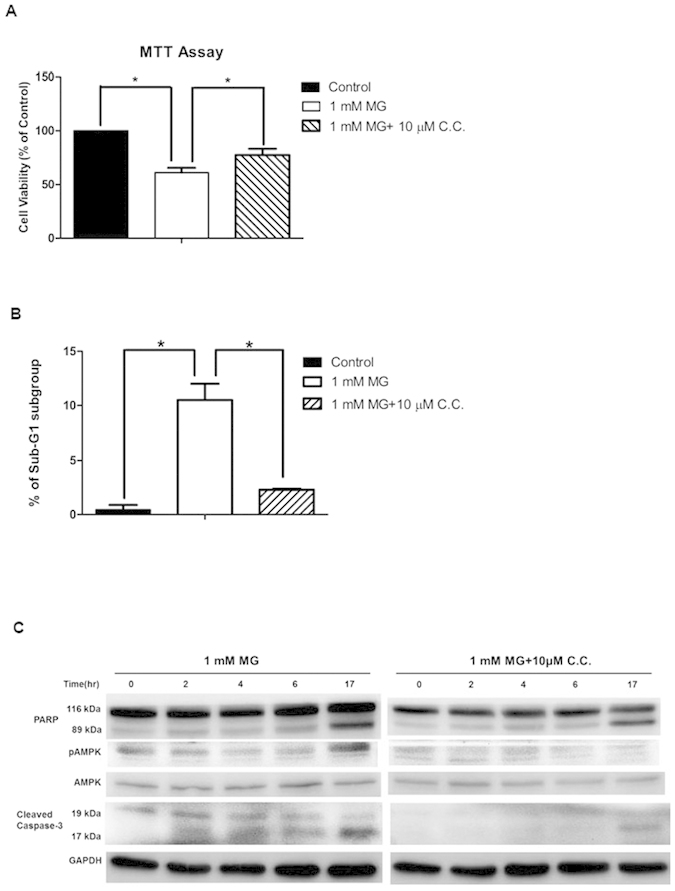
AMPK inhibitor partially rescues MG-induced cell death and apoptosis in RINm5F cells. Cells were treated in the presence or absence of 1 mM MG with or without compound C (C.C. 10 μM) in indicated time. (**A)** Cell viability was measured by MTT assay. Data are shown as relative cell viability (mean % ± S.E. bar) as compared with that in control (n = 5). *p < 0.05. (**B)** The percentage of apoptotic cells was calculated by measuring the percentage of cells in the sub-G1 population by using flow cytometry with propidium iodide (PI) staining (n = 3). *p < 0.05. (**C)** Western blot of PARP, p-AMPK/AMPK, cleaved caspase-3, and GAPDH as internal control.

We pre-treated cells with 10 μM compound C (a selective AMPK inhibitor) in MG-treated cells. Compound C partially rescued MG-induced cell death (Fig. 4A). Pre-treatment with 10 μM compound C significantly decreased the sub-G1 fraction in cells incubated with MG (Fig. 4B).

Compound C significantly inhibited phosphorylation of AMPK (Fig. 4C) and partially reduced MG-induced activation of caspase-3 and cleavage of PARP (Fig. 4C).

### GLP-1, but not metformin, significantly ameliorated AMPK activator- or MG-induced cell death and apoptosis

**Figure 5:**
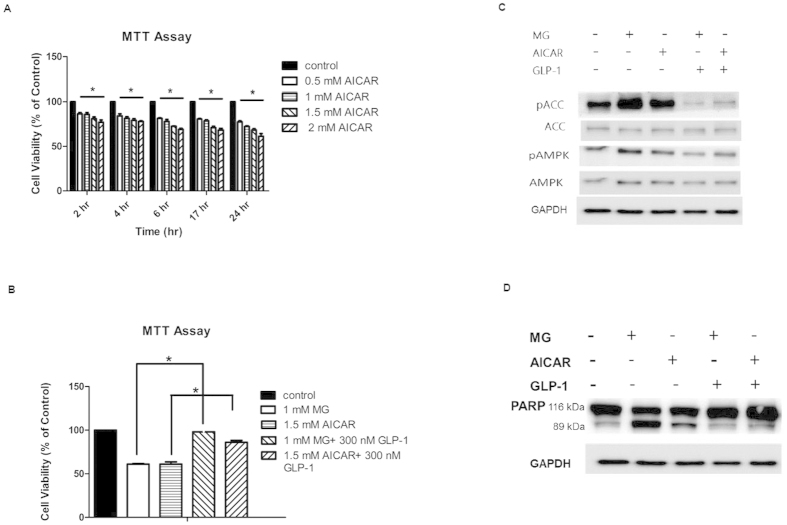
GLP-1 rescues AMPK activator AICAR-induced cell death, apoptosis and AMPK activation in RINm5F cells. (**A)** Cell viability was measured by MTT assay in different concentration of AICAR at indicated time. Data are shown as relative cell viability (mean % ± S.E. bar) as compared with that in control (n = 3). *p < 0.05. (**B)** Cell viability was measured by MTT assay in cells treated by 1 mM MG or 1.5 mM AICAR for 17 hr with or without pretreatment of 300 nM GLP-1 (n = 3). *p < 0.05. (**C)** Western blot of p-ACC/ACC and p-AMPK/AMPK in cells treated by 1 mM MG or 1.5 mM AICAR for 17 hr with or without pretreatment of 300 nM GLP-1. GAPDH was used as internal control. (**D)** Western blot of PARP in cells treated by 1 mM MG or 1.5 mM AICAR for 17 hr with or without pretreatment of 300 nM GLP-1. GAPDH was used as internal control. **(E)** Cell viability was measured by MTT assay in different concentration of metformin at indicated time. Data are shown as relative cell viability (mean % ± S.E. bar) as compared with that in control (n = 3). (**F)** Cell viability was measured by MTT assay in cells treated by 1 mM MG for 17 hr with pretreatment of different concentration of metformin. Data are shown as relative cell viability (mean % ± S.E. bar) as compared with that in control (n = 3).

We administered different concentrations of AMPK activator, 5-aminoimidazole-4-carboxamide ribonucleotide (AICAR, 0.5, 1, 1.5, and 2 mM) to RINm5F cells and then incubated for 2, 4, 6, 17, and 24 h, respectively. AICAR induced cell death in a dose- and time-dependent manner (Fig. 5A). GLP-1 significantly ameliorated both MG and AICAR-induced cell death, respectively (Fig. 5B). Furthermore, phosphorylation of ACC and AMPK, which indicates activity of AMPK, was significantly enhanced by the administration of MG and AICAR, but this phosphorylation was partially suppressed by GLP-1 (Fig. 5C). The degradation of PAPR was enhanced by MG and AICAR, but suppressed by GLP-1 (Fig. 5D).

Metformin, if not contraindicated, is the preferred first line pharmacological agent for type 2 diabetes^14^. Since some of the pleiotropic actions of metformin are associated with AMPK activation^15^, we examined if metformin treatment affects beta cell survival. As shown in Fig. 5E, we found that metformin, at different concentrations and treatment durations, did not induce RINm5F cell death. To further address the issue of whether metformin can prevent MG-induced cell apoptosis, we pre-incubated RINm5F cells in different concentrations of metformin (10, 25, 50, and 100 μM) in the presence of 1 mM MG. We found that metformin could not prevent the beta cell death induced by MG (Fig. 5F).

### GLP-1 also prevents MG-induced apoptosis of INS-1 and MIN 6 cells in part through PKA and PI3 kinase pathway, and partially via improving mitochondrial function and suppressing prolonged AMPK activation

**Figure 6:**
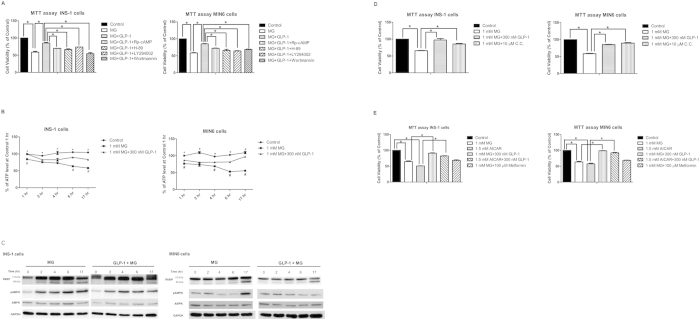
GLP-1 prevents MG-induced apoptosis of INS-1 and MIN6 cells in part through PKA and PI3 kinase pathway, and partially via improving mitochondrial function and suppressing prolonged AMPK activation. INS-1 and MIN6 cells were seeded overnight, and then were incubated with indicated treatment for indicated time. (A) INS-1 and MIN6 cells were treated in the absence of MG, 1 mM MG, 1 mM MG + 300 nM GLP-1, 1 mM MG + 300 nM GLP-1 + 100 μM Rp-cAMP, 1 mM MG + 300 nM GLP-1 + 10 μM H-89, 1 mM MG + 300 nM GLP-1 + 30 μM LY294002, 1 mM MG + 300 nM GLP-1 + 50 nM wortmannin, respectively. Cell viability was measured by MTT assay. Data are shown as relative cell viability (mean % ± S.E. bar) as compared with that in control (n = 3). *p < 0.05. (B) INS-1 and MIN6 cells were treated with or without 1 mM MG in the presence or absence of 300 nM GLP-1 for 1 hr, 2 hr, 4 hr, 6 hr, and 17 hr, respectively. Relative intracellular ATP concentration (%) compared with control in indicated time (n = 3). *p < 0.05 (Control vs. 1 mM MG at 1 hr, 2 hr, 4 hr, 6 hr and 17 hr), #p < 0.05 (1 mM MG vs. 1 mM MG + 300 nM GLP-1 at 1, 6, and 17 hr in INS-1 cells; 1, 4, 6, and 17 hr in MIN6 cells). (C) INS-1 and MIN6 were treated by 1 mM MG with or without GLP-1 for 0, 2, 4, 6, and 17 hr. Western blot of PARP, pAMPK/AMPK, and GAPDH was used as internal control. (D) INS-1 and MIN6 cells were treated in the absence or presence of 1 mM MG with or without 10 μM compound C (C.C.) for 17 hr. Cell viability was measured by MTT assay. Data are shown as relative cell viability (mean % ± S.E. bar) as compared with that in control (n = 3). *p < 0.05. (E) INS-1 and MIN6 cells were treated in the absence of 1 mM MG or 1.5 mM AICAR, 1 mM MG, 1.5 mM AICAR, 1 mM MG + 300 nM GLP-1, 1.5 mM AICAR + 300 nM GLP-1, 1 mM MG + 100 μM metformin for 17 hr, respectively. Cell viability was measured by MTT assay. Data are shown as relative cell viability (mean % ± S.E. bar) as compared with that in control (n = 3). *p < 0.05.

In order to verify and confirm the anti-apoptotic effect and molecular mechanisms of GLP-1 on MG-induced beta cell apoptosis, we further repeated some experiments in both INS-1 and MIN6 cells. Both PKA inhibitors (Rp-cAMP and H-89) and PI3 kinase inhibitors (LY294002 and wortmannin) partially suppressed the anti-apoptotic effect of GLP-1 on MG-induced apoptosis in both INS-1 and MIN6 cells (Fig. 6A). Steady decline in ATP production was observed in MG-treated INS-1 and MIN6 cells. Pretreatment of GLP-1 partially rescued ATP production in both cells (Fig. 6B).

Reduction of the intracellular ATP/AMP ratio has been shown to activate AMPK through phosphorylation of threonine 172 of α-subunit^22^. We also found that prolonged activation of AMPK was observed in MG-treated INS-1 and MIN6 cells. Pretreatment with GLP-1 reduced AMPK phosphorylation in MG-treated INS-1 and MIN6 cells (Fig. 6C). Pretreatment with GLP-1 also suppressed cleavage of PARP at different time point (Fig. 6C). To further verify the role of AMPK on MG-induced beta cell apoptosis, we pre-treated cells with 10 μM compound C (a selective AMPK inhibitor) in MG-treated INS-1 and MIN6 cells. Compound C partially rescued MG-induced cell death with a similar anti-apoptotic effect of GLP-1 (Fig. 6D). One of the AMPK activator, AICAR, induced cell death of INS-1 and MIN6 cells with a similar pro-apoptotic effect of MG (Fig. 6E). Pretreatment of GLP-1 in INS-1 and MIN6 cells rescued MG and AICAR-induced cell death (Fig. 6E). We also pretreated cells with another AMPK activator, metformin, in MG-treated INS-1 and MIN6 cells, and found that pretreatment with metformin neither aggravated nor rescued the MG-induced cell death (Fig.6E).

## Discussion

In this study, we provide the first evidence showing that GLP-1 rescues MG-induced beta cell apoptosis through improving mitochondrial function. Furthermore, we also found MG treatment induces prolonged AMPK activation and suppression of AMPK improved beta cell survival. Although metformin is known as an AMPK activator^15^, it neither induces beta cell apoptosis nor ameliorates MG-induced beta cell apoptosis.

The GLP-1 receptor agonist exendin-4 has been reported to inhibit hydrogen peroxide-induced apoptosis in MIN6 cells through a PKA and PI3K dependent pathway^19^. It also has been reported that GLP-1 prevents apoptosis of INS-1 cells in response to glucolipotoxicity or staurosporin through PI3K dependent pathway^20^,^21^. In this study, we also found both PKA inhibitors and PI3K inhibitors abrogated the anti-apoptotic effect of GLP-1 in RINm5F cells (Fig. 2A–D), INS-1 and MIN6 cells (Fig. 6A).

MG is an essential source of intracellular AGEs and abnormal accumulation of MG has been implicated in causing damage in various tissues and organs^3^. It has been reported that glycation of mitochondrial targets remarkably influences mitochondrial function in several studies. For example, MG decreased oxygen consumption of isolated mitochondria from rat kidney^23^. Also, MG or glyoxal reduced mitochondrial membrane potential, suppressed the activities of respiratory chain complexes, decreased ATP production, and elevated reactive oxygen species (ROS) levels^24^,^25^,^26^. Several lines of evidence suggest that mitochondrial dysfunction induces impairment of beta cell function and contributes to the pathogenesis of type 2 diabetes^27^,^28^. In this study, we found MG treatment lead to decreased oxygen consumption rate and intracellular ATP levels in RINm5F cells (Fig. 3A), INS-1 and MIN6 cells (Fig. 6B), and GLP-1 recovered mitochondrial function in MG-treated RINm5F cells (Fig. 3B). Fan *et al.* reported that human islet amyloid polypeptide inhibited mitochondrial biogenesis, and treatment with exendin-4 partially restored it^10^. Another study also showed exendin-4 significantly reduced oxidative stress and apoptotic cells through restoring the cytokine-induced reduction of electron transport chain proteins of mitochondria^11^. Tsuboi *et al.* reported that GLP-1 potentiates the mobilization of intracellular Ca^2+^ and stimulates mitochondrial ATP synthesis in MIN6 cells^29^. The above reports supported our finding on the anti-apoptotic effect of GLP-1 in MG-induced apoptosis through restoring the mitochondrial function of beta cells.

Reduction of the intracellular ATP/AMP ratio activates AMPK activity^22^. As revealed in this study, MG treatment led to prolonged activation of AMPK in RINm5F cells (Fig. 3C), INS-1 and MIN6 cells (Fig. 6C). Pretreatment with GLP-1 reduced AMPK phosphorylation in MG-treated RINm5F cells (Fig. 3C), INS-1 and MIN6 cells (Fig. 6C). So far, the role of AMPK in the survival of beta cells is controversial. A recent study demonstrated that rosiglitazone protects islet beta cells against palmitate-induced cell death by activating autophagy through activation of AMPK and subsequent inhibition of the mammalian target of rapamycin (mTOR) signaling pathway^30^. Another study showed that treatment of interleukin 1 or nitric oxide transiently and relatively early (12–24 hr) activated AMPK in insulinoma cells and rat islets. Activation of AMPK promotes the functional recovery of oxidative metabolism in beta cells and mitigates apoptosis induced by nitric oxide^31^. However, a few studies demonstrated that sustained activation of AMPK can trigger apoptosis of pancreatic beta cells^32^,^33^,^34^,^35^. One study showed that GLP-1 protected pancreatic beta cells against glucosamine-induced cytotoxicity through suppressing AMPK activity, and subsequently led to recovery of the phosphorylation levels of P70S6K and S6RP and alleviate glucosamine-induced cell death^36^. In our study, treatment with AMPK inhibitor compound C rescued MG-induced cell death and apoptosis in RINm5F cells (Fig. 4A–C), INS-1 and MIN6 cells (Fig. 6D). Consistently, treatment with AMPK activator AICAR also induced sustained AMPK activation (Fig. 5C), cell death and apoptosis in RINm5F cells (Fig. 5A,B,D), INS-1 and MIN6 cells (Fig. 6E). These results were compatible with the detrimental role of AMPK in beta cell survival in previous reports^32^,^33^,^34^,^35^,^36^. The discrepancy between different studies may be due to different durations of AMPK activation. Transient activation of AMPK can inhibit protein synthesis through phosphorylation of elongation factor-2 kinase^37^ and suppression of the mTOR pathway^38^, thus enhancing the restoration of cellular energy homeostasis and alleviating the burden of the unfolded nascent polypeptides^31^. However, prolonged activation of AMPK by AICAR has been reported to trigger beta cell apoptosis^32^,^33^. Several mechanisms have postulated to explain the link between AMPK and apoptosis. First, activation of AMPK may initiate cell cycle arrest in the G1 phase through inducing phosphorylation of the tumor suppressor p53^39^,^40^. While this arrest was reversible in the short term, but persistent activation of AMPK caused cell death^40^. Second, several studies showed that activation of AMPK triggers apoptosis through either a JNK- and caspase-3-dependent pathway^41^, or via suppression of protein kinase B pathway and mTOR-dependent protein synthesis, respectively^40^,^42^. Third, activation of AMPK is also associated with increased production of mitochondrial superoxide-derived radicals (ROS) and decreased activity of mitochondria^33^,^43^.

Metformin has been reported to activate AMPK and protect human islets against lipotoxicity^16^. Metformin has also been reported to prevent human pancreatic islets from impairment of glucose-stimulated insulin secretion induced by high glucose in human pancreatic islets^17^. These protective effects on beta cells are considered to exert through alleviating gluco- and lipo-toxicity indirectly, but the direct positive effect of metformin on β-cells is not generally accepted^44^. In this study, metformin neither induced cell death nor rescued MG-induced cell death in RINm5F cells (Fig. 5E,F), INS-1 and MIN6 cells (Fig. 6E). This indicated metformin had no anti-apoptotic effect against MG-induced beta cell apoptosis. It is well known that metformin primarily inhibits hepatic gluconeogenesis and improves insulin action on muscle and adipose tissue to lower high blood glucose^45^,^46^. In this study, we showed GLP-1, but not metformin, rescued MG-induced beta cell apoptosis. Therefore, clinical validation of the effect of adding GLP-1 to metformin treatment in improving beta cell survival and delaying disease progression is warranted. In a clinical trial, the GLP-1 analogue liraglutide added to metformin significantly lowered HbA1c in comparison with metformin alone^47^. The ongoing clinical trial, the Restoring Insulin Secretion (RISE) Consortium, is testing whether placebo, metformin alone, liraglutide plus metformin, or insulin (3 months) followed by metformin (9 months) can preserve or improve beta cell function in individuals with early type 2 diabetes or prediabetes^48^.

**Figure 7:**
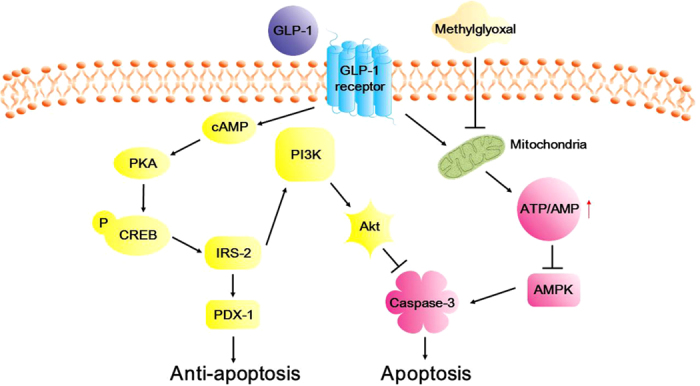
Schematic representation of the effect of GLP-1 against methylglyoxal (MG) toxicity in the beta cell. MG suppresses mitochondria function and leads to decrease ATP/AMP ratio, which in turn steadily activates AMPK, and the activation of AMPK subsequently leads to apoptosis. GLP-1 improved mitochondria function and in turn increases ATP/AMP ratio, which leads to inhibit sustained AMPK activation induced by MG, and subsequently suppress MG-induced apoptosis. In parallel, GLP-1 also exerts the anti-apoptotic effect through activation of PDX-1 in a PKA-dependent pathway. GLP-1 also activates PI3K/Akt pathway and inhibits caspase-3 activity and leads to suppress apoptosis.

In conclusion, we demonstrated for the first time that GLP-1 protects beta cells from apoptosis through improving mitochondrial function via suppressing sustained AMPK hyper-activation induced by MG (see model in Fig. 7). These data suggest inhibition of prolonged AMPK activation may improve the survival of beta cells under the condition of glucotoxicity. We also showed that GLP-1 protects beta cells from apoptosis in part through PKA and PI3K-dependent pathway, which are consistent with previous results^19^,^20^,^21^ (see model in Fig. 7).

## Methods

### Materials and chemicals

RPMI, dipeptide L-alanyl-L-glutamine, glucose, HEPES, sodium bicarbonate, sodium pyruvate, and 1X Gibco^®^ Antibiotic-Antimycotic for cell culture were from Thermo Fisher Scientific (Waltham, MA). Hoechst 33342, Annexin V, and PI were also from Thermo Fisher Scientific (Waltham, MA). Fetal bovine serum was from Biological Industries (Kibbutz Beit Haemek, Israel). MG, MTT, GLP-1, LY294002, and wortmanin were from Sigma-Aldrich, Inc. (St. Louis, MO). AMPK inhibitor compound C was from Merck Research Laboratories (Rahway, NJ). AICAR and metformin were from Cayman Chemical (Ann Arbor, MI). Rp-cAMP and H-89 were from Calbiochem® of Merck Millipore (Darmstadt, Germany).

### Cell line and cell culture

RINm5F, a rat insulinoma cell line, was purchased from the Bioresource Collection and Research Center (Hsinchu, Taiwan) and has been tested to prove free of mycoplasma contamination. RINm5F cells (passages 28–40) were grown in RPMI containing 10% (vol/vol) fetal calf serum at 37 °C with 5% CO_2_. RINm5F cells were seeded overnight. The next day, the 80% confluent cells were pre-incubated with GLP-1, or compound C or metformin for 1 h, and then MG was incubated for 17 hr. At the end of the experiment, MTT assay, Annexin V/Hoechst 33342 staining, flow cytometry, and cell lysate collection were performed.

INS-1, a rat insulinoma cell line was purchased from AddexBio Technologies (San Diego, CA) and has been tested to prove free of mycoplasma contamination. INS-1 cells were grown in RPMI containing 10% (vol/vol) fetal calf serum, 10 mM HEPES, 2 mM L-glutamine, 1 mM sodium pyruvate, and 0.05 mM 2-mercaptoethanol at 37 °C with 5% CO_2_. The protocol for experiments was the same as that in RINm5F cells.

MIN6 cells, kindly provided by S. Seino (Kobe University, Japan), were used between passages 15 and 25 and grown in DMEM containing 10% (vol/vol) fetal calf serum, 10 mM HEPES, 2 mM L-glutamine, 1 mM sodium pyruvate, and 0.05 mM 2-mercaptoethanol at 37 °C with 5% CO_2_. The protocol for experiments was the same as that in RINm5F cells at 37 °C with 5% CO_2_.

### MTT assay

RINm5F, INS-1, and MIN6 cells were seeded in a 24-well plate with 500 μl medium, and the above described chemicals were added. After the treatment, MTT assay was performed as described previously^49^.

### Staining for annexin V and Hoechst 33342

RINm5F cells were cultured in a 24-well plate. Staining for annexin V was performed as described previously^49^. The nuclear morphology of the cells was studied using the cell-permeable DNA-specific dye Hoechst 33342. Then, Hoechst 33342 was added at a final concentration of 10 μg/ml, and incubated for another 10 min at 37 °C^49^. The stained cells were then examined using a Zeiss AxioCam, CCD camera attached to the Zeiss Axiovert 200 M inverted microscope with fluorescence/phase or DIC imaging (Carl Zeiss Light Microscopy GmbH, Gottingen, Germany) and processed with MetaMorph® Software (Molecular Devices Inc., Sunnyvale, CA).

### Measurement of early and late apoptosis using annexin V and PI flow cytometry

We used FITC Annexin V Apoptosis Detection Kit with PI (BioLegend, San Diego, CA) to quantitate the early and late apoptosis of RINm5F cells. The protocol was described briefly according to the manufacturer’s protocol. RINm5F cells were treated with indicated chemicals for 17 hr, and then the cells were trypsinized and washed cells twice with cold BioLegends’ cell staining buffer, and then the cells were resuspended in Annexin V binding buffer at a concentration of 1 × 10^7^ cells/ml. One hundred μl of cell suspension was transferred to a 5 ml test tube, and then 5 μl of FITC Annexin V and 10 μl of Propidium Iodide (PI) solution were added. The cells were gently vortexed and incubated for 15 min at room temperature (25 °C) in the dark. Four hundred μl of Annexin V binding buffer was added to each tube and finally analyzed using a BD FACSVersse^TM^ flow cytometer (Becton, Dickinson and Company, San Jose, CA).

### Measurement of sub-G1 population by flow cytometry

RINm5F cells were trypsinized and then fixed in 80% ethanol at −20 °C for 30 min. The fixed cells were permeabilized with 0.5% Triton X-100 at room temperature for 5 min^50^. Then, these permeabilized cells were stained with 25 μg/ml PI for 10 min and finally analyzed using a BD FACSVersse^TM^ flow cytometer (Becton, Dickinson and Company, San Jose, CA) with an argon laser tuned to the 488 nm line for excitation.

### Extracellular flux (XF) analysis

Seahorse XF analyzer (Seahorse Bioscience, Billerica, MA) was employed to simultaneously measure the flux of oxygen (oxygen consumption rate) and protons (extracellular acidification rate) of adherent cells in a microplate over time. RINm5F cells were seeded in XF 24-well cell culture microplates (Seahorse Bioscience, Billerica, MA) at 1.0 × 10^5^ cells/well in 100 μl complete RPMI-1640 growth medium, and then incubated at 37 °C/5% CO_2_ for 18-20 h. Assays were initiated by replacing growth medium with 650 μl of low-buffered RPMI-1640 growth medium without sodium bicarbonate, HEPES, and containing only 1% FBS. The cells were incubated at 37 °C without 5% CO_2_ for 1 h to allow media temperature and pH to reach equilibrium before the first rate measurement. The following steps were performed according to the manufacturer’s instructions.

### ATP assay

The ViaLight® Plus Kit (Lonza Walkersville Inc., Rockland, ME) was used to examine the intracellular ATP levels; a decline of the ATP standard indicates the functional loss of mitochondria. RINm5F, INS-1, and MIN6 cells were seeded in a 96-well luminescence-compatible plate, and then were treated as described above. Fifty μl of Cell Lysis Reagent was added to each well. After at least 10 min, 100 μl of AMR PLUS was added to each well and the plate was cultivated for 2 min at room temperature. We then laid plate in an illuminometer and started running the project.

### Western blot

Equal amounts (50 μg) of RINm5F, INS-1, and MIN6 cell lysate were analyzed by Western blotting with rabbit anti-AMPK monoclonal antibody#ab32047 (Epitomics® Technology, Berlingame, CA), rabbit anti-phospho-AMPKα(Thr172) monoclonal antibody #2531 (Cell Signaling Technology, Beverly, MA), rabbit anti-PARP polyclonal antibody#9542S, anti-cleaved caspase-3, phospho-CREB/CREB, PDX1, phospho Akt/Akt and GAPDH (Cell Signaling Technology, Beverly, MA), respectively.

### Statistical Analyses

All results are presented as means ± standard error of mean (S.E.M.) of at least 3 independent and separate experiments. The differences between groups were calculated by Wilcoxon rank sum test (non-parametric statistics). All statistical analyses were carried out with SPSS 16.0 version (Chicago, Illinois). A p-value less than 0.05 was considered statistically significant.

## Additional Information

**How to cite this article**: Chang, T.-J. *et al.* Glucagon-like peptide-1 prevents methylglyoxal-induced apoptosis of beta cells through improving mitochondrial function and suppressing prolonged AMPK activation. *Sci. Rep.* **6**, 23403; doi: 10.1038/srep23403 (2016).
