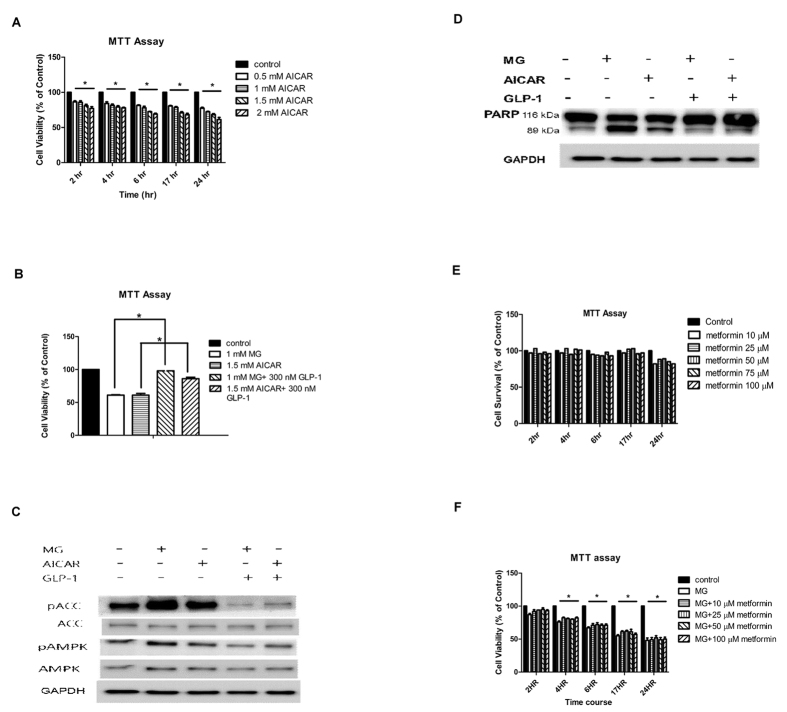# Corrigendum: Glucagon-like peptide-1 prevents methylglyoxal-induced apoptosis of beta cells through improving mitochondrial function and suppressing prolonged AMPK activation

**DOI:** 10.1038/srep26917

**Published:** 2016-05-31

**Authors:** Tien-Jyun Chang, Hsing-Chi Tseng, Meng-Wei Liu, Yi-Cheng Chang, Meng-Lun Hsieh, Lee-Ming Chuang

Scientific Reports **6**: Article number: 2340310.1038/srep23403; published online: 03212016; updated: 05312016

In this Article, Figure 5E and 5F were omitted. The Figure legends are correct. The correct Figure 5 appears below as Figure 1.

## Figures and Tables

**Figure 1:**